# Decrease in Bat Diversity Points towards a Potential Threshold Density for Black Cherry Management: A Case Study from Germany

**DOI:** 10.3390/plants8090320

**Published:** 2019-09-02

**Authors:** Jonas Geschke

**Affiliations:** Institute of Plant Sciences, University of Bern, Altenbergrain 21, 3013 Bern, Switzerland; jonas.geschke@ips.unibe.ch

**Keywords:** bioacoustic monitoring, bats, biological indicators, invasive species, sustainable forestry, close-to-nature forest management, nature conservation, *Prunus serotina* Ehrh., Chiroptera, *Pipistrellus* spp., *Myotis* spp., *Plecotus* spp.

## Abstract

In times of land use changes towards more close-to-nature forestry, the application of bioindicators becomes an interesting tool for effective land-use management schemes. Forest managers are increasingly confronted by alien tree species. Therefore, this case study aimed to investigate the influence of the invasive black cherry (*Prunus serotina*) on bats (Chiroptera: Verpertilionidae) in pine (*Pinus sylvestris*) forest ecosystems, in order to identify the potential of bats as bioindicators for a black cherry invasion. In three pre-classified succession stages of the black cherry, the diversity and relative abundance of bats were bioacoustically monitored for a period of 60 nights. From the bat call recordings made during the study period, eight bat species could be identified to species level. Within the succession stages of pine monoculture and light black cherry forest, a comparable bat diversity of eight bat species and three sonotypes with a similar relative abundance were observed. In dense black cherry forest, only four species and one sonotype were detected. Compared to the pine monoculture and light black cherry forest, the overall abundance of the bat community was significantly lower in the dense black cherry forest. Upon evaluation, those bat species associated with the edge and narrow space forager guilds were found to have a high sensitivity to a dense black cherry understory within naturally monocultural pine stands. Their activity patterns indicate that the transition from light to dense black cherry understory can be considered as a potential threshold value for a close-to-nature black cherry understory density in high canopy pine forest stands.

## 1. Introduction

Forests represent one of the most extensive ecosystems in the world. Their importance in tackling climate change and biodiversity loss, thus supporting landscape restoration and human wellbeing, is acknowledged by both science and policy [1,2,3,4]. At the same time, forests are degraded and converted to other forms of land use, as well as threatened by incoming alien species [5,6,7,8].

In Germany, around one-third of the land surface is covered by forest, of which approximately 60% is dominated by conifers and 40% by deciduous tree species [9,10]. While in the whole of Germany spruce (*Picea abies*) outweighs pine (*Pinus sylvestris*), with around 25% vs. 22% of the total forest cover [10], the forests of northeastern Germany are dominated by monocultural pine forests with 70–77% regional forest cover [10,11]. However, due to monocultural conifer forests having a sensitivity to disturbances and a low importance for biodiversity, recent forestry practices tend towards more close-to-nature silviculture [12,13]. This results in a higher rate of mixed forests and a higher diversity of vegetation structures within forest stands [14,15], which also benefits alien species [12,16]. Therefore, alien species must be managed so they do not suppress native species, which may cause severe changes to the functional diversity of forests. Except for the loss of local biodiversity and the alteration of for example forest regeneration patterns and soil conditions, the invasion risks of a range of incoming alien tree species are not fully assessed yet [17].

Certain tree species are tolerated and managed by the forestry sector, as they are more resistant to disease and climate variation than local species and thus economically beneficial [18]. The black cherry (*Prunus serotina*), however, is difficult to manage due to its high invasion potential through bird and mammal dispersion and high management costs [19,20]. After its first introduction to Europe for cultivation in parks (similar to other tree species [17]), the black cherry was used for improving soil conditions in pine monocultures [20,21]. Due to a lack of competition pressure in the pine understory and its high dispersal rates, it became an invasively spreading species, causing a range of ecological problems [20,22,23,24]. Existing studies on the black cherry in European forests mostly analyse its effects on soil conditions and understory vegetation [25,26,27,28,29,30]; however, its effects on different animal species remain to a large extent unknown.

In order to start filling this knowledge gap, this case study aimed to investigate the effects of increasing black cherry understory structures on bats (Chiroptera: Verpertilionidae). Bats are a group of highly specialised species and extraordinarily sensitive in terms of habitat structures and changes within such. European bats are strictly insectivorous and hold a high trophic level. According to their feeding behaviour, bat species can be allocated to different functional guilds: open space foragers (OSF), edge space foragers (ESF), and narrow space foragers (NSF) (as illustrated by [31,32]). The presence of bat species belonging to different functional guilds is highly correlated to the complexity of habitat features and vegetation structures within the different spaces and the availability of insects as a feeding resource [33,34,35,36,37,38]. Bats therefore combine a diverse range of habitats with the potential to correlate changes in bat diversity and abundance with different anthropogenic and biotic factors of landscape changes. Hence, they are good bioindicators of environmental chances [39,40,41,42], and their application as such can yield valuable information for bat-friendly forest management as well as nature conservation. Additionally, due to technical developments and improvements in scientific methods, bats can increasingly be monitored non-invasively and automatically.

**Figure 1:**
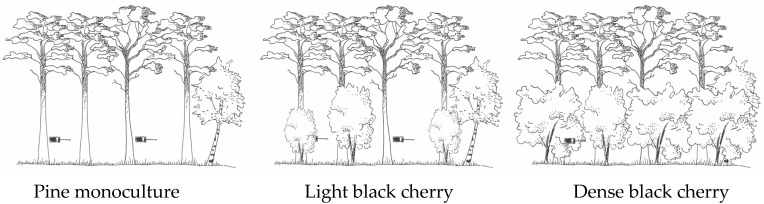
Classification of forest types representing different stages of the black cherry (*Prunus serotina*) invasion: Pine (*Pinus sylvestris*) monoculture with 0–5% black cherry (*Prunus serotina*) ground coverage, light black cherry with 6–60% black cherry ground coverage and dense black cherry with 61–100% black cherry ground coverage. Per forest type, two batcorder devices were used for the bioacoustic monitoring of bats. Drawings © Johanna Geschke.

In this case study, three black cherry succession stages, characterised by an increased black cherry density, were taken to represent a fictional black cherry invasion (Figure 1). The final goal was to identify bat species that are best applicable as bioindicators for close-to-nature black cherry management. Based on this, recommendations for sustainable forestry management schemes and nature conservation strategies are drawn.

## 2. Results

### 2.1. The Key Results in Brief

1. The bat diversity and relative abundance did not significantly differ between the pine (*Pinus sylvestris*) monoculture forest and the light black cherry (*Prunus serotina*) forest.
2. Nevertheless, different effects of the transition from the pine monoculture forest to the light black cherry forest on different bat genera and species were found:
  - While the overall activity of *Pipistrellus pygmaeus* (belonging to ESF) did not significantly differ between the pine monoculture and the light black cherry forest, a relatively high rate of feeding activities of *Pipistrellus pygmaeus* in the light black cherry forest indicated a positive effect of the black cherry, compared to the pine monoculture forest (here, no feeding activities were detected). The overall activity of both *Pipistrellus pipistrellus* (belonging to ESF) and *Pipistrellus nathusii* (belonging to ESF) was significantly affected by the black cherry density increase from the pine monoculture to the light black cherry forest: *Pipistrellus nathusii* was affected positively; *Pipistrellus pipistrellus* was affected negatively.
  - The overall activity of *Myotis nattereri* (belonging to NSF) and *Myotis daubentonii* (belonging to NSF) did not differ significantly between the pine monoculture and the light black cherry forest. However, a decrease of feeding activities of both *Myotis nattereri* and *Myotis daubentonii* indicated a negative effect of the black cherry. For *Myotis daubentonii*, no feeding calls were detected in the light black cherry forest.
  - The overall activity of the sonotype ‘Plecotus’ (belonging to NSF; see Section 4.3 for the definition of sonotypes) significantly increased in the light black cherry forest compared to the pine monoculture forest.
3. Compared to both the pine monoculture and the light black cherry forest, the diversity and relative abundance of all bat species detected significantly dropped in dense black cherry forest. A black cherry understory ground coverage of more than 60% was detected as a potential threshold value.

### 2.2. In Detail: From Plot to Forest Type Level

**Figure 2:**
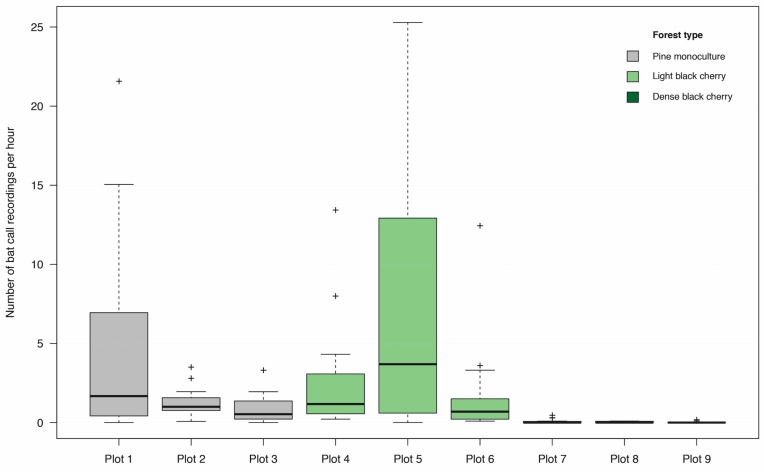
Number of bat call recordings per hour per plot: On the *x*-axis, the nine different study plots are listed; the *y*-axis is the scale for the number of recorded calls per hour. Plots 1–3 are characterised as pine (*Pinus sylvestris*) monoculture forest, plots 4–6 as light black cherry (*Prunus serotina*) forest, and plots 7–9 as dense black cherry forest.

A total of 3846 bat call recordings were made during the study period. Due to 66 recordings containing calls from more than one species, a total dataset of 3914 bat call recordings was analysed (Supplementary Materials Tables S2 and S3). Comparing the different plots per forest type, plots 1 and 5 had a noticeably higher frequency of bat call recordings than the others (Figure 2). As detected during the observation walks, this was due to a flight corridor next to plot 1 and the presence of a timber stack next to plot 5. Therefore, the differences between the plots were assumed to represent standard variability. This was endorsed by plot 4 having the highest bat diversity (Shannon index value of 2.06; Table 1), followed by plots 1‒3 as well as plots 5‒7. Plot 8 (1.31) had the lowest bat diversity not equal zero. Plot 9 had the most extreme Shannon index value (0), as here only one species (*Myotis nattereri*) was recorded. As plot 6 is located directly next to it, the very local forest type gradient from plot 6 to plot 9 in regard of the bat diversity was detected as a rapid transition.

As evident in these results on plot level, the effects of the black cherry on bat diversity and abundance differ depending on the black cherry understory density. Thus, the focus is placed on the generalised levels of the different forest types and the functional bat guilds per forest type.

Throughout the forest type gradient, the total recorded bat call length (≙100%) as a measure of bat activity was distributed by 36.14%, 63.45%, and 0.41%. Further overall monitoring per forest type results are summarised in Table 2.

**Figure 3:**
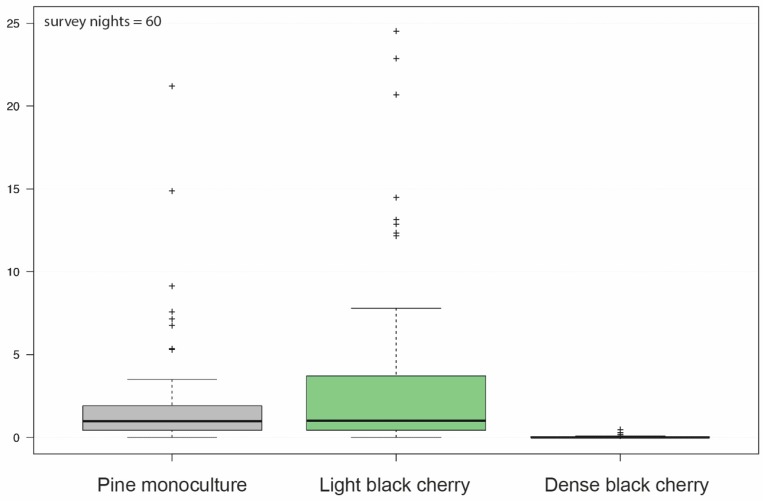
Number of bat call recordings per hour per forest: On the *x*-axis, the three different forest types pine (*Pinus sylvestris*) monoculture, light black cherry (*Prunus serotina*) and dense black cherry are listed. The *y*-axis represents the scale for the number of recorded bat calls per hour.

Similar to Figure 2, looking at the number of bat call recordings per hour on forest type level (Figure 3), the pine monoculture forest and the light black cherry forest did not differ significantly (*p* = 0.44). Both the pine monoculture and the light black cherry forest, however, differed significantly from the dense black cherry forest (both *p* < 0.001).

In the pine monoculture forest, eight species and three sonotypes were present during the study period: *Myotis myotis* (3.47%), *Myotis nattereri* (4.46%), ‘whiskered bats’ (1.03%), *Myotis daubentonii* (2.01%), ‘Plecotus’ (0.95%), ‘Nyctaloid’ (26.38%), *Eptesicus serotinus* (29.03%), *Nyctalus noctula* (4.85%), *Pipistrellus pipistrellus* (14.47%), *Pipistrellus pygmaeus* (7.09%), and *Pipistrellus nathusii* (6.26%). This results in a Shannon index of 1.92.

In the light black cherry forest, the same species diversity was identified with a different relative abundance: *Myotis myotis* (4.21%), *Myotis nattereri* (3.35%), ‘whiskered bats’ (0.81%), *Myotis daubentonii* (4.34%), ‘Plecotus’ (1.15%), ‘Nyctaloid’ (24.50%), *Eptesicus serotinus* (35.77%), *Nyctalus noctula* (8.61%)*, Pipistrellus pipistrellus* (6.06%), *Pipistrellus pygmaeus* (4.62%) and *Pipistrellus nathusii* (6.54%). This results in a Shannon index of 1.89.

In the dense black cherry forest, four species and one sonotype were detected: *Myotis myotis* (4.83%), *Myotis nattereri* (48.12%), ‘Nyctaloid’ (22.69%), *Eptesicus serotinus* (21.62%) and *Nyctalus noctula* (2.74%). This results in a Shannon index of 1.26.

**Figure 4:**
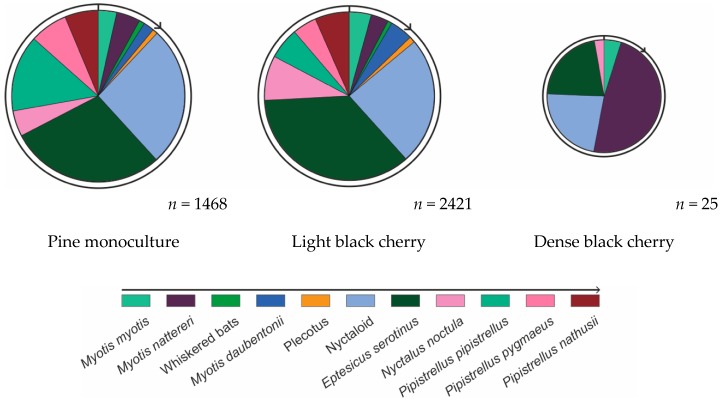
Relative abundance of bat species/sonotypes per forest type. The size of the pie charts is relative to the Shannon index of the respective forest type (1.92 vs. 1.89 vs. 1.26). The percentage values per species are provided in the main article text.

The above numbers are visualised in Figure 4. In the Supplementary Materials, Figure S3 shows the number of bat call recordings per hour per species in a comparable scale throughout the three forest types, and Tables S4–S6 provide more details to the above given diversity measurements.

Last but not least, considering the functional guilds of bats, the predominant guild was the NSF with 57.63% in the pine monoculture and 64.85% in the light black cherry forest. The second most abundant guild was the ESF, with 29.97% in the pine monoculture and 20.24% in the light black cherry forest. The OSF had the lowest relative abundance in both forest types, with 12.4% in the pine monoculture and 14.91% in the light black cherry forest.

Taking into account the number of bat call recordings per hour (Table 3), the presence of both the OSF and the ESF did not significantly differ between the two forest types (*p*_OSF_ = 0.83; *p*_ESF_ = 0.97). The NSF, in contrast, showed a significant increase in bat call recordings per hour in the light black cherry forest compared to the pine monoculture forest (*p*_NSF_ = 0.05). In more detail, the sonotype ‘Plecotus’ (belonging to NSF) significantly increased in terms of the number of recorded bat calls per hour from the pine to the light black cherry forest (*p* = 0.04). Similarly, *Pipistrellus nathusii* (belonging to ESF) significantly increased in the number of recorded bat calls per hour (*p* = 0.03). *Pipistrellus pipistrellus* (belonging to ESF), in contrast, significantly decreased in the number of recorded bat calls per hour from the pine monoculture to the light black cherry forest (*p* = 0.04). All other species and sonotypes detected in this study did not differ significantly between the pine monoculture and the light black cherry forest (*Myotis daubentonii*: *p* = 0.08; *Myotis nattereri*: *p* = 0.11; ‘Nyctaloid’: *p* = 0.19; *Myotis myotis*: *p* = 0.24; *Eptesicus serotinus*: *p* = 0.36; *Pipistrellus pygmaeus*: *p* = 0.44; ‘whiskered bats’: *p* = 0.53; *Nyctalus noctula*: *p* = 0.79).

In the transition from the light to the dense black cherry forest, the ESF disappeared. Thus, the relative abundance of bat guilds in the dense black cherry forest was 52% OSF versus 48% NSF. Considering the total number of recordings per hour, the detection of all three bat guilds significantly decreased, compared to the pine monoculture and light black cherry forests (all *p* < 0.001).

## 3. Discussion

### 3.1. Effects of Increasing Black Cherry Understory Structures on Bats

The overall goal of this case study was to investigate the effects of the invasive black cherry’s (*Prunus serotina*) density on bats in high canopy pine (*Pinus sylvestris*) forest stands. The aim was to assess the potential of bats to serve as bioindicator species for ecosystem changes caused by a black cherry invasion. Throughout a forest type gradient representing a fictional black cherry invasion, the presented results show a significant decrease in bat diversity and thus reveal a threshold density as a potential reference for effective black cherry management.

On a community level, the bat diversity did not significantly change between the pine monoculture and the light black cherry forest. On a functional guild level, the NSF showed a significant activity increase in the transition from the pine monoculture to the light black cherry forest. This is a comparable result to a case study by Rodríguez-San Pedro and Simonetti [43], but may especially be due to the high number of feeding calls recorded in the light black cherry plot 5, caused by a timber stack providing an increased abundance of insects [44] and thus better foraging opportunities [45]. Both the OSF and the ESF showed an activity increase within this transition; however, it was not significant. In the transition from the light to the dense black cherry forest, both the bat diversity and relative abundance significantly decreased. The high canopy pine forest stands with a dense black cherry understory were generally avoided by bats.

The increase in bat abundance and activity patterns in the light black cherry forest stands confirms the positive effects of forest understory structures [38,46,47,48,49]. However, it is also shown that the black cherry as an invasive species has negative impacts on its understory plant communities and has the ability to suppress native plant species [21,22,23]. Changes in soil condition caused by the black cherry [27,28] and a reduced light availability in black-cherry-invaded forest stands [27] decrease the density and natural regeneration of native ground vegetation species [21]. Nowakowska and Halarewicz [50] and Schilthuizen et al. [51] found the black cherry changing the local diversity and abundance of insect species. Therefore, as bats are highly specified foragers of certain insect species [32], it is most likely that not only the understory density is a factor but also the type of understory species (native or alien/invasive). As the black cherry is shown to change forest understory structures in short time periods [23,25,26,27], this study therefore showed similar results to those of Kusch et al. [52], Caras and Korine [53], and Müller et al. [35]: while understory vegetation structures are generally beneficial, the abundance of bats might be negatively affected by too-high densities in the forest understory vegetation. In this study, this was proven to be the case for a black cherry understory of more than 60% ground coverage.

### 3.2. Recommendations for a Close-to-Nature Black Cherry Management

Landscape managers handling pine forest ecosystems, either for forestry or for conservation purposes, should tolerate the black cherry in the forest understory and monitor its density in order to keep the management costs low. When deciding to combat the black cherry’s spread with respect to a high and close-to-nature bat diversity, this study indicates that a threshold value of 60% maximum ground coverage of the black cherry can be considered as a potential reference. As summarised in Section 2.1, bat species associated with the edge and narrow space forager guilds (in this study: genera *Pipistrellus, Myotis* and *Plecotus*) are best applicable as bioindicators for this threshold value. Once the black cherry understory density gets higher, individual black cherry trees should be removed from the forest stand in order to maintain a high complexity of vegetation structures as well as a close-to-nature understory ruggedness in order to give native plant species the chance to complement a diverse and jagged understory vegetation.

By considering both the understory density reference and the opportunity to use automated monitoring of bat species as described, forest management schemes have great potential to satisfy the needs of both forestry and nature conservation. While the economic benefit of pine forests remains stable and is supported by the regular thinning of individual black cherry trees, close-to-nature bat diversity is supported. Thereby, the ecological value of the forests increases.

### 3.3. Prospects for Future Studies

Future research into the effects of a black cherry invasion on bats or animal communities in general should look at a more differentiated classification of the black cherry density and at the potential effects of the vertical structure a black cherry understory brings to a forest stand. Also, monitoring of the insect availability and diversity in dependence on the black cherry density as well as monitoring of the light conditions (lumens) in different stages of the black cherry invasion should be conducted.

## 4. Materials and Methods

### 4.1. Study Area

The study was conducted on the grounds of the former estate of Linde (12°39′51.14″ East and 52°32′41.08″ North) and its close surroundings of the Havelland in the German federal state of Brandenburg, northeastern Germany (Figures S1 and S2). The Havelland is a mosaic landscape characterised by human settlements, both intensive and extensive agriculture, forestry, protected areas, and wetlands. The study area is dominated by pine forest stands. Based on a detailed vegetation mapping [54], the black cherry density within the study area was assessed and—for feasibility reasons—divided into three classes: pine (*Pinus sylvestris*) monoculture forest with 0‒5% black cherry (*Prunus serotina*) coverage, light black cherry forest with 6‒60% black cherry ground coverage, and dense black cherry forest with 61‒100% black cherry ground coverage, respectively (see Figure 1). With a minimum distance of 365 m, 796 m, and 842 m, the distance between two plots of the same forest type was kept as small as possible in order to have similar site conditions throughout the study sites. The plots had a size of 100 m × 100 m.

### 4.2. Bioacoustic Monitoring of Bats

The bioacoustic monitoring of bat species was conducted using batcorder devices (ecoObs GmbH, Nuremberg, Germany). Each plot centre was assigned two random locations for the batcorder devices (Table S1). The monitoring was designed to cover a total of 60 nights from 30 July to 27 September 2016. Each night, one randomly chosen plot per forest type was surveyed, resulting in a repetition of 20 samplings per plot. The recording of bat calls was done from one hour before local sunset until one hour after local sunrise the next day. The batcorders were set to the default settings recommended by the manufacturer. Additionally, observation walks were conducted in order to look at potential reasons for particular outliers in the recordings already made. Such observation walks took place from sunset until around 10 p.m. During the observation walks, a handheld bat detector (D240X Ultrasound Detector, Pettersson Elektronik AB, Uppsala, Sweden) was used for the monitoring. In order to identify representatives of the edge and narrow space foraging guilds (e.g., *Pipistrellus* and *Myotis* species), the focus was given to a frequency range of 45 ± 10 kHz.

The recorded bat calls [55] were analysed in the software bcAdmin (Version 3.6; ecoObs GmbH, Nuremberg, Germany) in combination with bcAnalyze (Version 3 Pro; ecoObs GmbH) and bcIdent (Version 1.5; ecoObs GmbH). The activity of bats was differentiated into transfer calls, final buzz feeding calls, and social calls. Automatic bat species identifications by bcIdent with at least 95% probability were judged statistically correct, except for species that were not known to be present or known to be specifically rare in the study area. Those and all bat calls identified with less than 95% probability were manually re-identified. Different studies from the literature were used as guidance for manual species identification [56,57,58,59]. For the species identification, the bioacoustic threshold values of the start, main/peak, and end frequency of the bat calls were consulted. In the end, bat calls of *Myotis myotis*, *Myotis nattereri*, *Myotis daubentonii*, *Eptesicus serotinus, Nyctalus noctula, Pipistrellus pipistrellus*, *Pipistrellus pygmaeus*, and *Pipistrellus nathusii* were identified. Bat calls that were impossible to determine to species level but had a similar call structure and frequency range were assigned to the sonotypes ‘Plecotus’ (*Plecotus auritus* and *Plecotus austriacus*), ‘whiskered bats’ (*Myotis mystacinus* and *Myotis brandtii*), and ‘Nyctaloid’ (*Eptesicus serotinus*, *Eptesicus nilssonii*, *Vespertilio murinus*, *Nyctalus leisleri*, and *Nyctalus noctula*—these are the Nyctaloid species potentially occurring in the study area [60,61]). For the functional analysis, the OSF is represented by *Eptesicus serotinus*, *Nyctalus noctula*, and the sonotype Nyctaloid. The ESF is represented by the pipistrelle species *P. pipistrellus*, *P. pygmaeus*, and *P. nathusii*. The NSF is represented by *Myotis myotis*, *Myotis nattereri*, and *Myotis daubentonii* as well as the sonotypes ‘whiskered bats’ and ‘Plecotus’.

### 4.3. Monitoring of Microclimatic Site Conditions

The microclimatic site conditions were monitored with HOBO dataloggers of the type Pro v2 U23-001 (Onset Computer Corporation, Bourne, MA, USA, Firmware 3.2.0). Temperature and relative humidity [62] were recorded in the same spatial and temporal sampling design as the bat calls. The minimum temperature throughout the study period was 4.51 °C (in a pine monoculture stand) and the maximum temperature was 28.49 °C (in a dense black cherry stand). The mean temperatures throughout the forest type gradient were 14.47 °C, 14.57 °C, and 15.13 °C. The relative humidity ranged from 35.17% (in a dense black cherry stand) to 100%; the mean relative humidity in the forest type gradient was 86.83%, 87.50%, and 85.63%. No significant differences between the microclimatic conditions in the forest types or plots were found. As such, the microclimatic site conditions in this study were judged to have no effect on the bat diversity or relative abundance.

### 4.4. Data Analysis

All statistical analyses, both for the bat and the climate data, were conducted using the software RStudio (RStudio Inc, Boston, MA, USA, Version 0.99.879) and R (R Foundation for Statistical Computing, Vienna, Austria, Version 3.3.1). Similar to the automatic bat call identification, the significance threshold was set to alpha = 5%. All numbers and values were rounded to two decimal places. The non-parametric Mann‒Whitney U test was used to test the bat and climate data for significant differences. The relative abundance of bats was calculated based on the bat call length per forest type or species/sonotype. The median value is provided to describe the bat data, as it is independent from outlier values. For further interpretation of the bat data, the Shannon‒Weaver index [63,64] was used to describe the general bat diversity respective to the relative abundance of the bat species/sonotypes.

## Figures and Tables

**Table 1:** Number of bat call recordings per hour and Shannon-Weaver index per plot. Species: pine (*Pinus sylvestris*); black cherry (*Prunus serotina*).

Pine Monoculture			Light Black Cherry			Dense Black Cherry		
	rec/h	H’		rec/h	H’		rec/h	H’
Plot 1	89.15 median 1.67	1.69	Plot 4	48.25 median 1.17	2.06	Plot 7	1.28 median 0	1.27
Plot 2	25.06 median 1.00	1.92	Plot 5	137.83 median 3.69	1.73	Plot 8	0.53 median 0	1.31
Plot 3	16.33 median 0.53	2.00	Plot 6	31.56 median 0.69	1.84	Plot 9	0.37 median 0	0

rec/h: Bat call recordings per hour (*n* = 20); *H’*: Shannon‒Weaver index.

**Table 2:** Overall monitoring results per forest type. Distribution of recorded bat call length, number of identified bat species/sonotypes, bat call recordings per hour and Shannon‒Weaver index. Species: pine (*Pinus sylvestris*); black cherry (*Prunus serotina*).

	Pine Monoculture	Light Black Cherry	Dense Black Cherry
Distribution of Recorded Bat Call Length	36.14%	63.45%	0.41%
Number of Identified Bat Species/Sonotypes	8 species and 3 sonotypes	8 species and 3 sonotypes	4 species and 1 sonotype
Bat Call Recordings Per Hour (*n* = 60)	128.64 median 0.98	213.42 median 1.01	2.08 median 0
Shannon‒Weaver index	1.92	1.89	1.26

**Table 3:** Number of total bat call recordings and bat call recordings per hour per bat guild and forest type. The arrows indicate an increase or decrease in bat activity; the star symbolises that the change was significant. The data was gathered in 60 survey nights. Species: pine (*Pinus sylvestris*); black cherry (*Prunus serotina*).

	Pine Monoculture		Light Black Cherry		Dense Black Cherry
Open Space Foragers (OSF)	*n* = 846; rec/h: 76.39 median 0.25	⬈	*n* = 1570 rec/h: 143.91 median 0.23	*⬊	*n* = 12 rec/h: 1.09 median 0
Edge Space Foragers (ESF)	*n* = 440; rec/h: 38.70 median 0.44	⬈	*n* = 490 rec/h: 42.48 median 0.34	*⬊	*n* = 0
Narrow Space Foragers (NSF)	*n* = 182 rec/h: 15.45 median 0.17	*⬈	*n* = 361 rec/h: 31.26 median 0.28	*⬊	*n* = 13 rec/h: 1.08 median 0

*n:* Total number of bat call recordings; rec/h: Bat call recordings per hour; median: Single recording length where 50% of the total number of recordings were shorter and 50% longer.

## Supplementary Materials

The following are available online at https://www.mdpi.com/2223-7747/8/9/320/s1, the supplementary material is grouped into 1. information on the study area and 2. information on the gathered bioacoustical data, Figure S1: Location of the study area ‘Linde’, Figure S2: Distribution of study sites throughout the study area, Table S1: Batcorder location coordinates, Table S2: General results of species/sonotype identification, Table S3: Number of bat call recordings per hour per species per forest type, Figure S3: Number of bat call recordings per hour per species in comparable scale, Table S4: Bat diversity and relative abundance in the pine (*Pinus sylvestris*) monoculture forest, Table S5: Bat diversity and relative abundance in the pine (*Pinus sylvestris*) forest with light black cherry (*Prunus serotina*) in the understory, Table S6: Bat diversity and relative abundance in the pine (*Pinus sylvestris*) forest with dense black cherry (*Prunus serotina*) in the understory.
