# Supplementary material for: Decrease in Bat Diversity Points towards a Potential Threshold Density for Black Cherry Management: A Case Study from Germany
Source: Plants (Basel). 2019 Sep 2;8(9):320. doi: 10.3390/plants8090320 (PMC6784187; doi:10.3390/plants8090320)
Supplement: Supplementary file 1 [file plants-08-00320-s001.pdf]

Supplementary Material

Decrease in Bat Diversity Points towards a Potential Threshold Density for Black Cherry Management: A Case Study from Germany

Jonas Geschke

Institute of Plant Sciences, University of Bern, Altenbergrain 21, 3013 Bern, Switzerland; jonas.geschke@ips.unibe.ch

The supplementary material is grouped into 1. information on the study area and 2. information on the gathered bio-acoustical data. One figure/table is shown per page.

---

1. Information on the study area

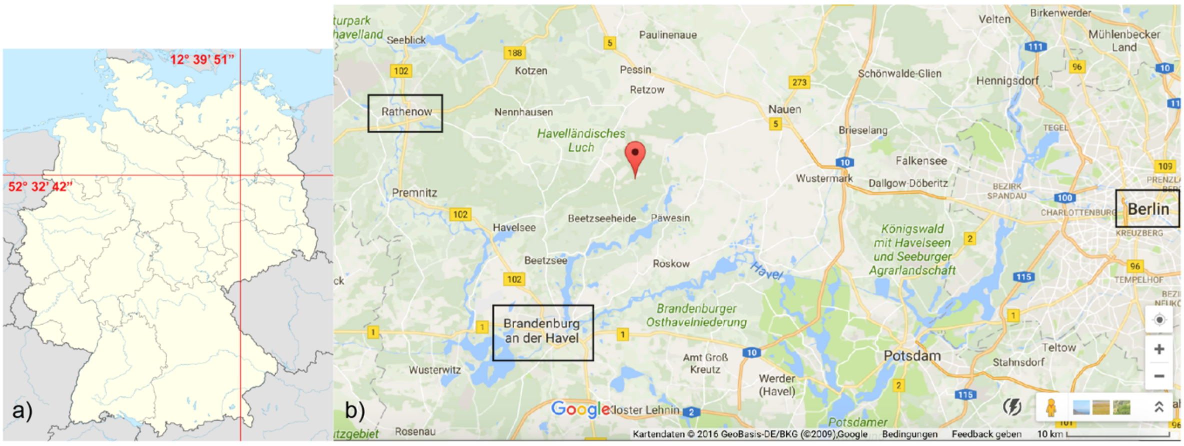

Figure S1. Location of the study area 'Linde': **a**) North-eastern location within Germany; **b**) Location on regional scale: The study area is surrounded by the cities of Rathenow in the west, Brandenburg an der Havel in the south and Berlin in the east.
Sources: a) URL upload.wikimedia.org/wikipedia/commons/e/ed/Germany_adm_location_map.svg – edited by author; b) GeoBasis-DE/BKG © 2016 Google – edited by author

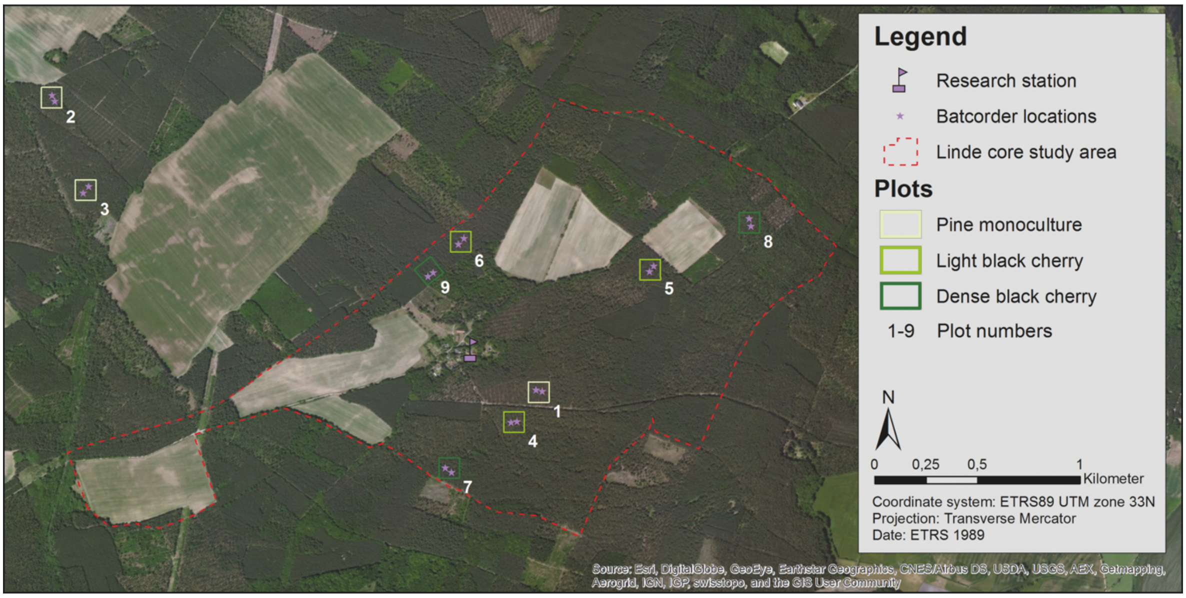

Figure S2. Distribution of study sites throughout the study area: The research station Linde is located at 12°39'51,314" East and 52°32'41,908" North. Plots 1 and 4-9 are located within the Linde core study area, the plots 2 and 3 in the close surroundings north-eastern of the core study area. Plots 1-3 are classified as pine (*Pinus sylvestris*) monoculture, plots 4-6 as light black cherry (*Prunus serotina*) and plots 7-9 as dense black cherry.
Source: Aerial picture © ESRI Inc, California, United States

Table S1. Batcorder location coordinates.
Species: pine (*Pinus sylvestris*); black cherry (*Prunus serotina*)

Forest type	Plot	Batcorder 1	Batcorder 2
Pine monoculture	1	E 12°40′10.227“ ǀ N 52°32′35.823“	E 12°40′8.615“ ǀ N 52°32′35.97“
	2	E 12°38′1.62“ ǀ N 52°33′19.12“	E 12°38′1.056“ ǀ N 52°33′20.095“
	3	E 12°38′9.814“ ǀ N 52°33′4.79“	E 12°38′11.381“ ǀ N 52°33′5.756“
Light black cherry	4	E 12°40′3.999“ ǀ N 52°32′30.908“	E 12°40′2.386“ ǀ N 52°32′30.704“
	5	E 12°40′36.997“ ǀ N 52°32′55.18“	E 12°40′38.042“ ǀ N 52°32′56.115“
	6	E 12°39′48.727“ ǀ N 52°32′59.535“	E 12°39′47.331“ ǀ N 52°32′58.561“
Dense black cherry	7	E 12°39′47.495“ ǀ N 52°32′22.596“	E 12°39′45.767“ ǀ N 52°32′23.219“
	8	E 12°41′2.356“ ǀ N 52°33′4.082“	E 12°41′2.88“ ǀ N 52°33′2.855“
	9	E 12°39′41.042“ ǀ 52°32′53.992“	E 12°39′39.647“ ǀ N 52°32′53.34“

2. Information on the gathered bio-acoustical data

Table S2. General results of species/sonotype identification: Bat diversity and relative abundance based on the total of 3914 identified bat call recordings made during 60 survey nights.
The species/sonotype identification data used for the statistical analysis is marked bold. The relative abundance is based on the total bat call length. The data for the automatically identified 'Nyctaloid' species (software bcIdent; ecoObs GmbH, Nuremberg, Germany; Version 1.5) is calculated with the sonotype 'Nyctaloid' as reference. Only the top fife abundant species/ species groups are presented.

Sonotype / Species		Type of identification	Bat call recordings	Bat calls	Total bat call length [ms]	Relative abundance [%]
Myotis myotis		manual	**141**	**877**	**161.37**	**3.94**
Myotis nattereri		manual	**199**	**1012**	**160.99**	**3.93**
**'Whiskered bats'**	*Myotis mystacinus*<br>*Myotis brandtii*	manual	**23**	**270**	**36.42**	**0.89**
Myotis daubentonii		manual	**133**	**841**	**143.65**	**3.51**
**'Plecotus'**	*Plecotus auritus*<br>*Plecotus austriacus*	manual	**60**	**186**	**43.73**	**1.07**
**'Nyctaloid'**		manual	**1008**	**4918**	**1030.37**	**25.17**
	Nyctaloid (genera *Nyctalus*, *Vespertilio*, *Eptesicus*, *Tadarida* and *Vespertilio*)	automatic	311	1626	339.08	32.91
	Eptesicus nilssonii	automatic	236	1791	329.37	31.97
	Spec. (calls but no species identified)	automatic	347	800	229.52	22.28
	Nyctalus noctula	automatic	60	251	56.79	5.51
	Nycmi (*E. serotinus*, *V. murinus*, *N. leiseri*)	automatic	20	199	29.59	2.87
	Rest *(Myotis dasycneme, Barbastella barbastellus, E. serotinus, V. murinus, N. leisleri, Hypsugo savii, P. pipistrellus, P. pygmaeus, P. nathusii, P. kuhlii)*	automatic	34	251	46.03	4.47
Eptesicus serotinus		manual	**981**	**7765**	**1362.24**	**33.28**
Nyctalus noctula		manual	**439**	**1153**	**295.7**	**7.22**
Pipistrellus pipistrellus		manual	**382**	**2031**	**371.55**	**9.08**
Pipistrellus pygmaeus		manual	**278**	**1106**	**225.05**	**5.50**
Pipistrellus nathusii		manual	**270**	**1226**	**262.34**	**6.41**

Table S3. Number of bat call recordings per hour per species per forest type.
The data was gathered in 60 survey nights. Species: pine (*Pinus sylvestris*); black cherry (*Prunus serotina*)

Species/nonotype	Pine monoculture	Light black cherry	Dense black cherry
Myotis myotis	3.88<br>median 0.00	8.34<br>median 0.00	0.09<br>median 0.00
Myotis nattereri	6.5<br>median 0.08	8.94<br>median 0.09	0.99<br>median 0.00
'Whiskered bats'	0.82<br>median 0.00	1.27<br>median 0.00	0
Myotis daubentonii	2.44<br>median 0.00	9.47<br>median 0.00	0
'Plecotus'	1.81<br>median 0.00	3.24<br>median 0.00	0
'Nyctaloid'	35.33<br>median 0.00	56.38<br>median 0.00	0.553<br>median 0.00
Eptesicus serotinus	29.88<br>median 0.00	60.10<br>median 0.08	0.47<br>median 0.00
Nyctalus noctula	11.18<br>median 0.08	27.43<br>median 0.08	0.07<br>median 0.00
Pipistrellus pipistrellus	19.70<br>median 0.20	13.81<br>median 0.08	0
Pipistrellus pygmaeus	11.11<br>median 0.00	13.08<br>median 0.04	0
Pipistrellus nathusii	7.89<br>median 0.00	15.59<br>median 0.09	0

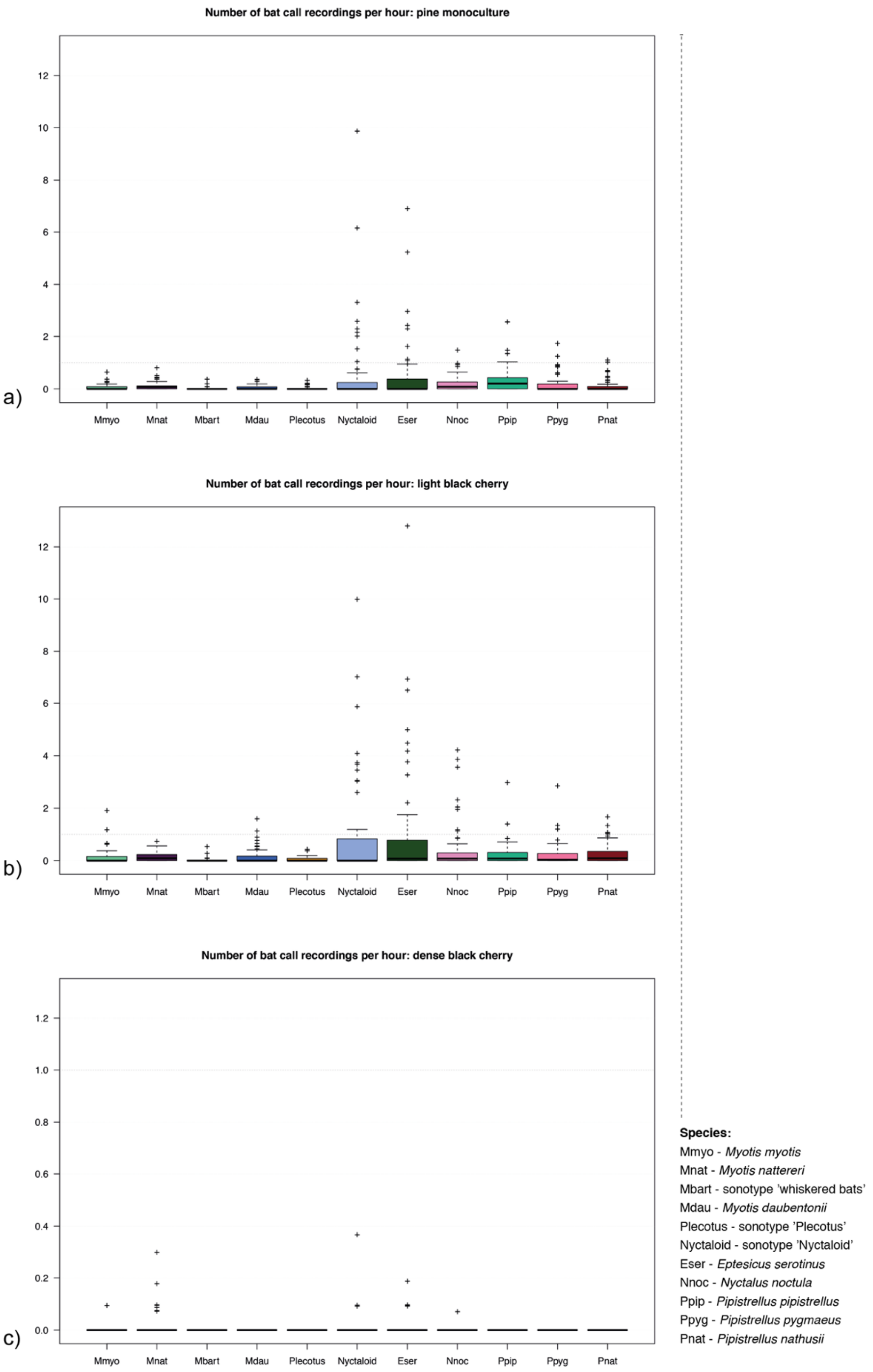

Figure S3. Number of bat call recordings per hour per species in comparable scale: **a**) Pine monoculture; **b**) Light black cherry; **c**) Dense black cherry. For comparability, the value of 1 bat call recording per hour is marked with a grey line.

Species: pine (*Pinus sylvestris*); black cherry (*Prunus serotina*)

Table S4. Bat diversity and relative abundance in the pine (*Pinus sylvestris*) monoculture forest. Monocultural pine ≙ 0 to 5% black cherry coverage. N = 60 survey nights. The relative abundance is based on the total bat call length.

Sonotype/Species	Bat call recordings	Bat calls	Total bat call length [ms]	Relative abundance [%]
Myotis myotis	**43**	**331**<br>median 5	**51.33**<br>median 1.03	**3.47**
Myotis nattereri	**81**	**440**<br>median 2	**65.95**<br>median 0.60	**4.46**
**'Whiskered bats'**	**9**	**118**<br>median 14	**15.31**<br>median 1.78	**1.03**
Myotis daubentonii	**27**	**205**<br>median 4	**29.79**<br>median 0.91	**2.01**
**'Plecotus'**	**22**	**56**<br>median 2	**13.98**<br>median 0.46	**0.95**
**'Nyctaloid'**	**385**	**1843**<br>median 3	**390.21**<br>median 0.79	**26.03**
Eptesicus serotinus	**325**	**2300**<br>median 5	**429.45**<br>median 0.16	**29.03**
Nyctalus noctula	**136**	**224**<br>median 1	**71.68**<br>median 0.47	**4.85**
Pipistrellus pipistrellus	**225**	**1170**<br>median 3	**214.09**<br>median 0.80	**14.47**
Pipistrellus pygmaeus	**126**	**508**<br>median 3	**104.95**<br>median 0.72	**7.09**
Pipistrellus nathusii	**89**	**456**<br>median 4	**92.58**<br>median 0.87	**6.26**

Table S5. Bat diversity and relative abundance in the pine (*Pinus sylvestris*) forest with light black cherry (*Prunus serotina*) in the understory.
Light black cherry ≙ 6 to 60% black cherry coverage. N = 60 survey nights. The relative abundance is based on the total bat call length.

Sonotype/Species	Bat call recordings	Bat calls	Total bat call length [ms]	Relative abundance [%]
Myotis myotis	**97**	**543**<br>median 5	**109.23**<br>median 1.03	**4.21**
Myotis nattereri	**106**	**535**<br>median 3.5	**86.91**<br>median 0.66	**3.35**
**'Whiskered bats'**	**14**	**152**<br>median 8.5	**21.11**<br>median 1.29	**0.81**
Myotis daubentonii	**106**	**636**<br>median 4	**113.87**<br>median 0.98	**4.34**
**'Plecotus'**	**38**	**130**<br>median 3	**29.75**<br>median 0.71	**1.15**
**'Nyctaloid'**	**617**	**3061**<br>median 3	**636.33**<br>median 0.74	**24.50**
Eptesicus serotinus	**651**	**5454**<br>median 7	**929.14**<br>median 1.34	**35.77**
Nyctalus noctula	**302**	**928**<br>median 1	**223.56**<br>median 0.46	**8.61**
Pipistrellus pipistrellus	**157**	**861**<br>median 3	**157.46**<br>median 0.71	**6.06**
Pipistrellus pygmaeus	**152**	**598**<br>median 2	**120.11**<br>median 0.61	**4.62**
Pipistrellus nathusii	**181**	**770**<br>median 2	**169.76**<br>median 0.69	**6.54**

Table S6. Bat diversity and relative abundance in the pine (*Pinus sylvestris*) forest with dense black cherry (*Prunus serotina*) in the understory.
Dense black cherry ≙ 61 to 100% black cherry coverage. N = 60 survey nights. The relative abundance is based on the total bat call length.

Sonotype/Species	Bat call recordings	Bat calls	Total bat call length [ms]	Relative abundance [%]
Myotis myotis	**1**	**3**	**0.82**	**4.83**
Myotis nattereri	**12**	**37** median 1.5	**8.12** median 0.52	**48.12**
**'Whiskered bats'**	**0**	**0**	**0**	**0**
Myotis daubentonii	**0**	**0**	**0**	**0**
**'Plecotus'**	**0**	**0**	**0**	**0**
**'Nyctaloid'**	**6**	**14** median 1.5	**3.83** median 0.60	**22.69**
Eptesicus serotinus	**5**	**11** median 2	**3.65** median 0.72	**21.62**
Nyctalus noctula	**1**	**1**	**0.46**	**2.74**
Pipistrellus pipistrellus	**0**	**0**	**0**	**0**
Pipistrellus pygmaeus	**0**	**0**	**0**	**0**
Pipistrellus nathusii	**0**	**0**	**0**	**0**
